# Sex specific effects of “junk-food” diet on calcium permeable AMPA receptors and silent synapses in the nucleus accumbens core

**DOI:** 10.1038/s41386-020-0781-1

**Published:** 2020-07-30

**Authors:** Yanaira Alonso-Caraballo, Tracy L. Fetterly, Emily T. Jorgensen, Allison M. Nieto, Travis E. Brown, Carrie R. Ferrario

**Affiliations:** 1 Neuroscience Graduate Program, University of Michigan, Ann Arbor, MI USA; 2 Department of Pharmacology, University of Michigan, Ann Arbor, MI USA; 3 Neuroscience Program, University of Wyoming, Laramie, WY USA; 4 Pharmaceutical Science, University of Wyoming, Laramie, WY USA

**Keywords:** Reward, Obesity, Motivation, Neurotransmitters

## Abstract

CP-AMPARs in the nucleus accumbens (NAc) mediate cue-triggered motivation for food and cocaine. In addition, increases in NAc CP-AMPAR expression and function can be induced by cocaine or sugary, fatty junk-foods. However, the precise nature of these alterations and the degree to which they rely on the same underlying mechanisms is not well understood. This has important implications for understanding adaptive vs. maladaptive plasticity that drives food- and drug-seeking behaviors. Furthermore, effects of junk-foods on glutamatergic plasticity in females are unknown. Here, we use a combination of protein biochemistry and whole-cell patch clamping to determine effects of diet manipulation on glutamatergic plasticity within the NAc of males and females. We found that junk-food consumption increases silent synapses and subsequently increases CP-AMPAR levels in males in the NAc of male rats. In addition, a brief period of junk-food deprivation is needed for the synaptic insertion of CP-AMPARs and the maturation of silent synapses in males. In contrast, junk-food did not induce AMPAR plasticity in females but may instead alter NMDAR-mediated transmission. Thus, these studies reveal sex differences in the effects of junk-food on NAc synaptic plasticity. In addition, they provide novel insights into how essential food rewards alter NAc function.

## Introduction

Activity in mesocorticolimbic circuits influences a range of motivational processes including the pursuit of food and drug rewards. There is general agreement that the nucleus accumbens (NAc) is a central hub in this network, integrating information from glutamatergic and dopaminergic inputs to direct behavior toward essential rewards like food and sex, as well as nonessential rewards like cocaine [1–3]. While it is well established that addictive substances like cocaine produce long-lasting alterations within the NAc, much less is known about how food rewards affect NAc function. In particular, understanding the potential effects of sugary, fatty junk-foods (JFs) on mesocorticolimbic systems and motivational processes has become increasingly important as the obesity epidemic continues to rise worldwide. Indeed, there is substantive evidence from both the human and preclinical literature that consumption of sugary, fatty foods alters NAc function in a manner that promotes the pursuit of food and overeating [4–6].

Glutamatergic plasticity within the NAc core is a key mediator of enhanced motivation for both food and cocaine [7–10]. For example, cue-triggered food seeking and the “incubation” of cocaine seeking after prolonged withdrawal are mediated by activation of high conductance calcium permeable-AMPA receptors (CP-AMPARs) within the NAc core [11, 12]. Furthermore, consumption of a sugary, fatty JF diet enhances NAc CP-AMPAR-mediated transmission and expression [13] and potentiates cue-triggered food seeking in males [14]. However, whether the processes mediating CP-AMPAR upregulation are ubiquitous or specific to JF vs. cocaine experience is unknown.

Increases in NAc core CP-AMPAR synaptic transmission mediate the incubation of cocaine craving, are persistent (lasting months), and require a relatively long drug free period (~3 weeks). During this drug free period, there is an accumulation of CP-AMPARs on the cell surface that is accompanied by their synaptic recruitment and retention [11, 15–17]. Furthermore, in the NAc shell, the synaptic insertion of CP-AMPARs in response to cocaine is preceded by increases in the number of silent synapses in the NAc (i.e., immature synapses containing NMDA receptors [NMDARs], but lacking AMPARs), which are thought to then mature via insertion of CP-AMPARs [18, 19]. Thus, following cocaine withdrawal and the development of incubation of craving there is an increase in functional mature synapses (shell) and an increase in CP-AMPAR insertion in the NAc (shell and core, [18, 19]).

On the other hand, consumption of JF for as few as 10 days increases NAc core CP-AMPAR synaptic transmission in males after just 1 day of JF deprivation, an effect that persists for at least 1 month [13]. However, whether a JF free period is necessary for CP-AMPAR upregulation, or whether plasticity of silent synapses precedes the insertion of CP-AMPARs following JF consumption is unknown. Furthermore, effects of JF on NAc glutamatergic transmission and AMPAR expression in females have not been determined. Therefore, studies below determined the effects of JF on NAc surface expression of GluA1 and GluA2 AMPAR subunits and glutamatergic synaptic transmission in the NAc core of males and females. Overall, we found sex specific effects, with JF enhancing CP-AMPAR transmission and inducing plasticity of silent synapses in males, but not females. In addition, a brief period of JF deprivation was needed for the synaptic insertion of CP-AMPARs and the maturation of silent synapses in males, but not for increases in CP-AMPAR surface protein expression. These results are discussed in consideration of established roles for CP-AMPARs in cue-triggered craving and enhanced motivation.

## Materials and methods

### Subjects

Male and female selectively bred obesity-prone and obesity-resistant rats were used. The original selection was conducted by Barry Levin from outbred Sprague Dawley rats [20]. Rats used here were bred in house (colonies maintained by the UM Breeding Core [21–23]), housed on a reverse 12-h light/dark cycle, had free access to food and water throughout, and were group housed unless otherwise noted. Rats were 60–70 days old and were weighed 3–4 times per week unless otherwise specified. Procedures were approved by The University of Michigan and The University of Wyoming Institutional Animal Care and Use Committee in accordance with AAALAC and AVMA guidelines. See also https://sites.google.com/a/umich.edu/ferrario-lab-public-protocols/ for additional details. Ns for all data are given in the “Results” section below. In scientific papers “N” is a common abbreviation for the number of subjects in a a study. Example, “10 people were in the control group, and 10 people were in the experimental group, thus the N was 10 per group”.

### JF and chow diet

Control groups were given free access to standard lab chow (Lab Diet 5LOD: 4 kcal/g; 4.5% fat, 23% protein, 48.7% carbohydrates; % of caloric content) in their home cage, whereas experimental groups were given free access to a JF diet made in house [13, 24]. The JF was a mash of Ruffle potato chips (40 g), Chips Ahoy (130 g), Nesquik (130 g), Jiff peanut butter (130 g), Lab Diet 5001 (200 g), and 180 ml of water (19.6% fat, 14% protein, and 58% carbohydrates; 4.5 kcal/g). For all studies, rats were assigned to JF or chow groups counterbalanced for initial weight within obesity-prone and obesity-resistant groups.

### Effects of JF on GluA1 and GluA2 surface expression in the NAc of male rats

We first determined the effect of 10 days of JF followed by 2 weeks of JF deprivation on AMPAR subunit surface expression. During the deprivation period, rats in the JF group were given free access to standard lab chow and JF was no longer available. Next, we used an established BS^3^-crosslinking procedure followed by western blotting to determine the surface and intracellular expression of GluA1 and GluA2 AMPAR subunits [12, 13, 25]******. Briefly, NAc tissue was extracted, chopped (400 μm), and incubated in aCSF containing BS^3^ (5 mM) for 30 min (4 °C). Glycine (100 mM) was added to stop the crosslinking reaction (10 min, 4 °C). Samples were centrifuged (2 min; 14,000 RPM; 4 °C and the pellet was resuspended in ice cold lysis buffer containing (in mM): 25 HEPES, 500 NaCl, 2 EDTA, 1 DTT, 1 PMSF, 20 NaF; 1:100 EDTA-free Protease Inhibitor Cocktail (Sigma-Aldridge; 11836170001); and 0.1% Nonidet P-40 [v/v]; pH 7.4) and homogenized by sonication. Samples were then stored at −80 °C. For SDS-PAGE and western blotting samples were heated (70 °C, 10 min) in Laemmli sample treatment buffer with 5% β-mercaptoethanol, loaded (20 µg protein), and electrophoresed on 6% gels made in house. Proteins were transferred onto PVDF membranes (GE Healthcare Amersham™ Hyperfilm™ Fisher Scientific; 45-001-505). Membranes were rinsed, blocked (1 h, RT, 5% [w/v] nonfat dry milk in TBS-Tween 20 [TBS-T; 0.05% Tween 20, v/v]), and incubated overnight (4 °C) with primary antibodies to GluA1 (Thermo Scientific; PA1-37776; 1:1000 in TBS) or GluA2 (EMD Millipore; AB1768-I; 1:2000 in TBS-T and 5% milk). Membranes were then washed in TBS-T, incubated with HRP-conjugated secondary (Invitrogen, Carlsbad, CA; 1 h, RT), washed, and immersed in chemiluminescence detecting substrate (Thermo Scientific; Pierce™ ECL Western Blotting Substrate Cat. no. 32106). Images were acquired on film and Ponceau S (Sigma-Aldrich) was used to determine total protein in each lane. Bands of interest were then quantified using Image J (NIH).

To determine the effects of JF deprivation on GluA1 and GluA2 surface expression, a separate cohort of obesity-prone (*n* = 26) and obesity-resistant (*n* = 23) male rats were separated into three groups: chow fed, JF no deprivation, and JF 24-h deprivation. The JF deprivation and no deprivation groups received JF for 10 days, while the chow group remained on chow throughout the study. The JF deprivation group received chow diet for 24 h before tissue collection and BS^3^-crosslinking, whereas the JF no deprivation remained on JF throughout. GluA1 and GluA2 surface expression were determined as described above.

### Effects of Junk-food on synaptic transmission in NAc core of males

CP-AMPAR-mediated transmission and silent synapses were evaluated in medium spiny neurons (MSNs) from obesity-prone male rats. Recordings were made from chow controls, and on day 10 of JF exposure or after 24–48 h of JF deprivation. This timing was chosen to improve feasibility of recordings, rather than timing all recordings to 24 h. Recordings from chow controls and JF groups were interspersed. MSNs were identified visually by cell body size (~15 μm in diameter) and by their capacitance and resting membrane potential after break in, as required recording conditions prevent the proper evaluation of membrane properties distinct to MSNs.

Established whole-cell patch clamping approaches were used [13, 26]. Briefly, coronal slices (300 μm) containing the NAc were made using a vibratory microtome (Leica Biosystems, Buffalo Grove, IL, USA) and allowed to recover in oxygenated aCSF prior to recording. Patch pipettes were pulled from 1.5 mm borosilicate glass capillaries (WPI, Sarasota, FL; 3–7 MΩ resistance). Evoked EPSCs (eEPSCs) were elicited by local stimulation (0.05–0.30 mA square pulses, 0.1 ms, delivered every 20 s) using a bipolar electrode placed ~300 μm lateral to recorded neurons. The minimum amount of current needed to elicit a synaptic response with <15% variability in amplitude was used. If >0.30 mA was required, the neuron was discarded. Pipettes were filled with cesium chloride (CsCl) internal solution containing (in mM): 140 CsCl, 10 HEPES, 2 MgCl2, 5 Na+-ATP, 0.6 Na+-ATP, 2 QX314, pH 7.3, 285 mOsm. eEPSCs were recorded at −70 mV before and after application of the CP-AMPAR selective antagonist Naspm (200 µM; as in [13, 15]) in the presence of the GABA_A_ receptor antagonist picrotoxin (50 µM). For measures of the AMPA/NMDA ratio, eEPSCs were recorded at +40 and −70 mV before and after bath application of NMDAR antagonist APV as previously described (50 µM; [27, 28]).

For silent synapse measures, recording conditions and solutions were as previously described [29, 30]. Pipettes were filled with CsCl internal solution containing (in mM): 117 CsCl, 2.8 NaCl, 5 MgCl2, 20 HEPES, 2 Mg2+ATP, 0.3 Na2+GTP, 0.6 EGTA, 0.1 spermine, and sucrose to bring osmolarity to 275–280 mOsm and pH to ~7.25. Minimal stimulation protocols were performed as previously described [29–31]. Briefly, the frequency of presynaptic stimulation was set at 0.33 Hz. After obtaining small (<40 pA) eEPSCs at −70 mV, stimulation intensity was reduced in small increments to the point where failures (no response) vs. successes (visible eEPSC) were clearly and visually distinctive. Stimulation intensity and frequency were kept constant for the remaining duration of the experiment. Following 50 sweeps at −70 mV, cells were gradually raised to +45 mV for 50 sweeps and then back down for a second set of 50 sweeps at −70 mV. Failures vs. successes were defined visually. The percentages of silent synapses were calculated using the equation: (1-Ln(F-70)/Ln(F+45)) × 100, in which F-70 was the failure rate at −70 mV and F+45 was the failure rate at +45 mV. All drugs and reagents were obtained from Sigma-Aldrich (St. Louis, MO).

### Effects of JF on GluA1 and GluA2 surface expression and synaptic transmission in the NAc of female rats

Studies in females were conducted alongside those in males but are described separately for the sake of clarity. We first determined the effects of 10 or 30 days of JF diet consumption followed by 2 weeks of JF deprivation on GluA1 and GluA2 surface expression using the BS^3^-crosslinking procedure described above. Separate cohorts with their own chow control groups were used for 10 days and 30 days studies. These durations and time points were chosen because they produce increases in GluA1 surface expression in males [13] and results below.

Separate cohorts of obesity-prone female rats were used to examine potential effects of 10 days of JF followed by 2 weeks of JF deprivation on CP-AMPARs and AMPA/NMDA ratio using the electrophysiological approaches described above. All recordings were conducted from slices prepared when females were in the metestrus/diestrus phase of the cycle. This phase was chosen because this is when motivation for food, food intake, and cue-triggered food seeking are highest in females [32–34]. Estrous cycle phase was determined by daily observations of vaginal epithelial cell cytology, precopulatory, and copulatory behaviors as previously described [34, 35]. Briefly, epithelial cells were collected at the same time each day by vaginal lavage during the dark phase and cell morphology was then used to determine cycle phase (Olympus CKX53, bright field). Body weight, food intake, and precopulatory and copulatory behaviors (such as ear wiggling, darting, and lordosis) were also used to further verify the estrous cycle phase.

### Statistics and data analysis

Two-tailed *t*-tests, one-way or two-way ANOVAs, and Sidak’s post-hoc multiple comparisons were used (Prism 8, GraphPad, San Diego, CA). Electrophysiology data were analyzed using Clampfit 10.4 (Molecular Devices). Comparisons were made between data collected within the same cohort of animals (i.e., given chow or JF side by side).

## Results

### Food intake and body weight

Weight gain and food intake analysis are summarized in Supplementary Tables 1 and 2, respectively. In males destined for biochemical studies, obesity-prone rats tended to be heavier overall and tended to gain more weight on the JF diet compared with obesity-resistant rats. However, these differences were not statistically significant (see Supplementary Table 1a for group means and two-way ANOVA comparing cumulative weight gained). For males destined for electrophysiological recordings, obesity-prone rats given the JF diet gained more weight than the chow group (Supplementary Table 1b). In addition, obesity-prone rats ate more than obesity-resistant rats (Supplementary Table 2a) and all animals fed a JF diet ate more than the chow groups (Supplementary Table 2).

In females destined for biochemical studies, obesity-prone rats gained more weight than obesity-resistant rats regardless of diet manipulation (Supplementary Table 1a). Furthermore, JF had no effect on weight gain in female rats (Supplementary Table 1). This is not unexpected considering the short duration of JF consumption, and the relative resistance of female rodents to diet-induced obesity (see also [23, 36] for additional discussion). In addition, for females destined for biochemical studies, obesity-prone rats ate more JF than obesity-resistant rats (Supplementary Table 2a). For all studies, female rats fed a JF diet ate more than the chow group (Supplementary Table 2).

### Effects of Junk-food diet on AMPAR subunit expression and synaptic transmission in males

#### Junk-food increases NAc GluA1 surface expression only in obesity-prone male rats

**Fig. 1:**
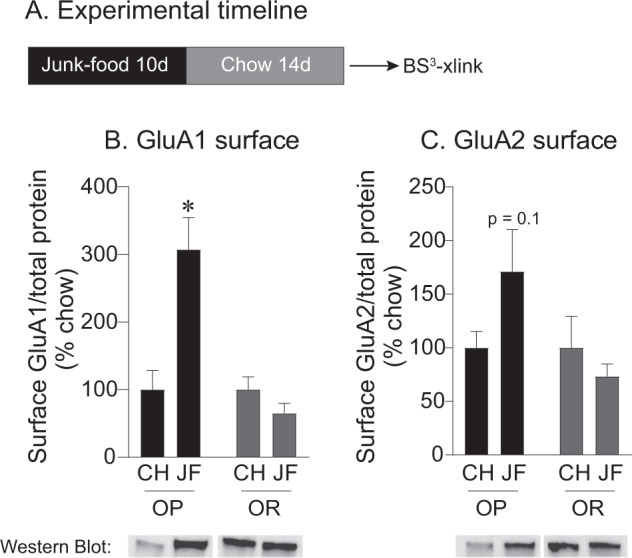
Junk-food increases NAc GluA1 surface expression only in obesity-prone male rats. **a** Experimental timeline. **b** Average GluA1 surface expression in obesity-prone (OP) and obesity-resistant (OR) rats. GluA1 surface expression increased following 2 weeks of junk-food deprivation in obesity-prone, but not obesity-resistant rats. **c** Average GluA2 surface expression in obesity-prone and obesity-resistant rats. GluA2 surface expression was not altered by junk-food in either group. All data shown as mean ± SEM unless otherwise noted. *p < 0.05 obesity-prone chow vs. *p* < 0.05 junk- food.

We first determined the effects of 10 days of JF diet followed by 2 weeks of withdrawal on GluA1 and GluA2 surface expression in obesity-prone and obesity-resistant male rats (see timeline Fig. 1a, *n* = 10 rats per group). GluA1 surface expression was increased in obesity-prone but not obesity-resistant rats following 2 weeks of JF deprivation (Fig. 1b: two-way ANOVA group × diet interaction: *F*_(1,36)_ = 16.1, *p* = 0.0003; main effect of group *F*_(1,36)_ = 16.1, *p *= 0.0003; Sidak’s multiple comparisons, OP-CH vs. OP-JF, *p* < 0.0001). GluA2 surface expression was not altered by JF in either group compared with controls (Fig. 1c; see also below). In addition, intracellular expression of GluA1 or GluA2 was not altered by JF in either group (data not shown; GluA1: two-way ANOVA no group × diet interaction: *F*_(1,36)_ = 0.22, *p *= 0.6; CH vs. JF: *F*_(1,36)_ = 0.01, *p *= 0.9; OP vs. OR: *F*_(1,36)_ = 1.4, *p *= 0.2; GluA2: two-way ANOVA no group × diet interaction: *F*_(1,38)_ = 3, *p *= 0.1; CH vs. JF: *F*_(1,38)_ = 0.5, *p *= 0.5; OP vs. OR: *F*_(1,38)_ = 0.1, *p *= 0.7). This pattern is consistent with an increase in surface expression of CP-AMPARs and is consistent with previous results [13].

#### Junk-food deprivation is not necessary for increased GluA1 surface expression in obesity-prone male rats

**Fig. 2:**
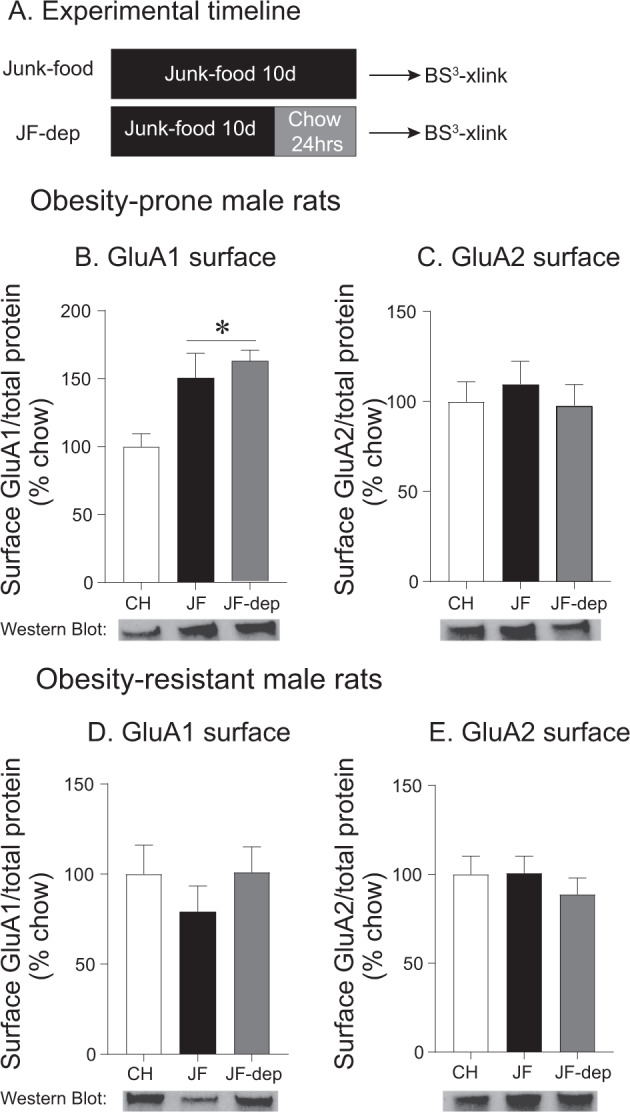
Junk-food increases GluA1 surface expression in obesity-prone males regardless of deprivation. **a** Experimental timeline. **b** Average surface expression of GluA1 in obesity-prone (OP) male rats. Similar increases in GluA1 surface expression were found following 10 days of junk-food consumption with and without junk-food deprivation. **c** Average surface expression of GluA2 in obesity-prone male rats. GluA2 surface expression was not altered by junk-food or junk-food deprivation. **d** Average surface expression of GluA1 in obesity-resistant (OR) male rats. No effects of junk-food or junk-food deprivation were found. **e** Average surface expression of GluA2 in obesity-resistant male rats. No effects of junk-food or junk-food deprivation were found. *p < 0.05 compared with chow.

Next, we determined if changes in surface expression of AMPAR subunits in the NAc require a period of JF deprivation (see timeline Fig. 2a, obesity prone: *n* = 26 and obesity resistant: *n* = 23). We found increases in GluA1 surface expression in both JF no deprivation and 24-h deprivation groups compared with controls (Fig. 2b: one-way ANOVA: *F*_(2,23)_ = 6.4, *p *= 0.006; Sidak’s multiple comparison CH vs. JF no deprivation, *p *= 0.005, CH vs. JF-dep, *p *= 0.02). Thus, a period of JF deprivation is not needed for increased GluA1 surface expression in obesity-prone males. In addition, JF did not affect GluA2 surface expression in either group (Fig. 2c). In contrast, JF did not affect GluA1 (Fig. 2d) or GluA2 (Fig. 2e) surface expression in obesity-resistant males.

#### Junk-food deprivation is required for increased synaptic CP-AMPAR-mediated transmission in obesity-prone male rats

**Fig. 3:**
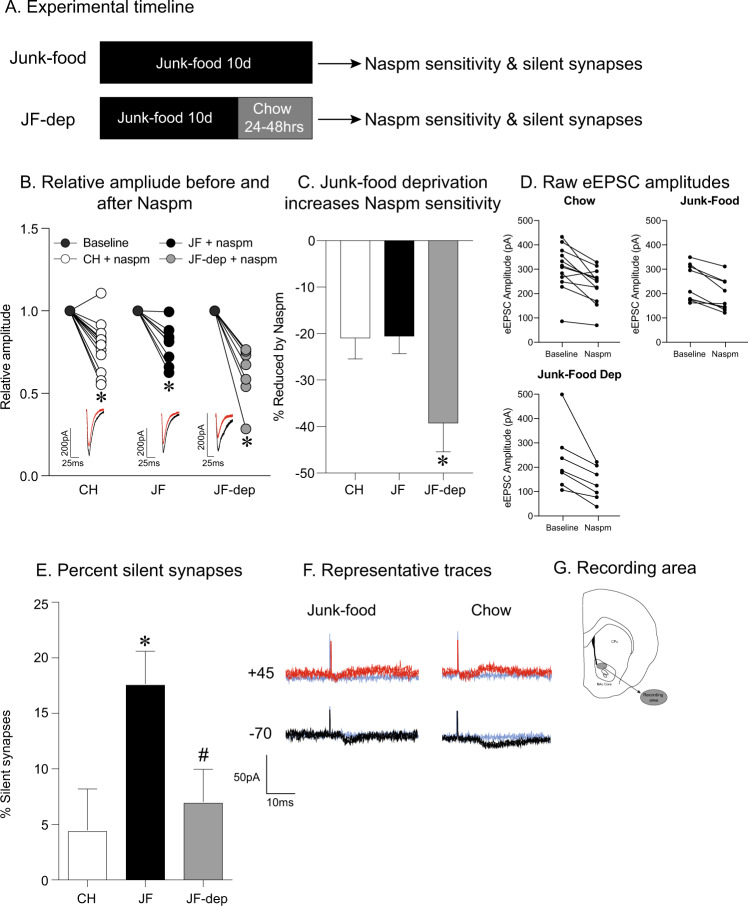
Effects of junk-food on CP-AMPAR-mediated transmission and generation of silent synapses in obesity-prone male rats. **a** Experimental timeline. CP-AMPAR-mediated transmission and measures of silent synapses were determined following 10 days of junk-food with and without 24–48 h deprivation. **b** Normalized eEPSC amplitude before and after Naspm (200 µM). Naspm produced similar decreases in eEPSC amplitude in obesity-prone (OP) chow and junk-food groups. However, junk-food deprivation enhanced Naspm sensitivity. The inset shows example traces from chow and junk-food groups before (black) and after bath application of Naspm (red). **c** Average reduction in eEPSC amplitude in chow and junk-food groups. Junk-food deprivation resulted in enhanced Naspm sensitivity. **d** Raw eEPSC amplitudes for chow, junk-food, and junk-food deprivation groups. Range of baseline amplitudes does not differ across groups. **e** The percent of silent synapses in obesity-prone (OP) chow, junk-food, and junk-food deprivation groups. **f** Representative traces of eEPSCs at +45 (NMDAR mediated) and −70 mV (AMPAR mediated) for junk-food and chow groups. *p < 0.05 chow vs. junk-food, ^#^*p* < 0.05 junk-food vs. junk-food deprivation. **g** Depiction of recording area within the NAc core.

Next, we used whole-cell patch clamping approaches to determine the contribution of CP-AMPARs to synaptic transmission in NAc core MSNs following 10 days of JF with and without 24-h JF deprivation (see timeline in Fig. 3a; chow: *n* = 11 cells, six rats; JF no deprivation: *n* = nine cells, five rats; JF 24-h deprivation: *n* = seven cells, five rats). For these studies only obesity-prone rats were used, as we have yet to see effects of diet manipulation on AMPAR protein expression or synaptic transmission in obesity-resistant rats (current results and [12, 13]). The CP-AMPAR selective antagonist, Naspm, produced a significant reduction in eEPSC relative amplitude in all groups (Fig. 3b: one-way ANOVA: *F*_(5,50)_ = 19.5, *p *= 0.02; Sidak’s multiple comparisons: baseline vs. chow, *p* < 0.001, baseline vs. JF no deprivation, *p *= 0.0004, baseline vs. JF dep, *p* < 0.0001). However, Naspm produced a significantly larger reduction in eEPSC amplitude in the JF 24-h deprivation group than either control or JF no deprivation groups (Fig. 3c: one-way ANOVA: *F*_(2,25)_ = 4.4, *p *= 0.02; Sidak’s multiple comparisons: chow vs. JF no deprivation, *p *= 0.99; chow vs. JF dep, *p *= 0.04; JF no deprivation vs. JF dep, *p *= 0.05). Furthermore, the magnitude of Naspm sensitivity was comparable in chow controls and the JF no deprivation group (Fig. 3c, *p *= 0.99). Thus, although surface CP-AMPAR protein expression is increased in both JF exposed groups, a period of JF deprivation is necessary for increased CP-AMPAR synaptic transmission.

#### Generation of silent synapses after junk-food exposure

The insertion of CP-AMPARs has been associated with the generation of silent synapses in the NAc shell following cocaine [37, 38]. Therefore, we determined the effects of JF with and without 24-h deprivation on the generation of silent synapses in the NAc core of obesity-prone rats following JF (chow: *n* = 14 cells, six rats; JF no deprivation: *n* = 11 cells, five rats; JF 24-h deprivation: *n* = five cells, four rats). There was a significant increase in the percentage of silent synapses following 10 days of JF without deprivation compared with controls (Fig. 3e: one-way ANOVA: *F*_(2, 27)_ = 13, *p *= 0.0001, Sidak’s multiple comparisons: chow vs. JF no deprivation, *p* < 0.05). Furthermore, the percentage of silent synapses following 24-h of JF deprivation was similar to that of chow fed controls (Fig. 3e: Sidak’s multiple comparison: CH vs. JF dep, *p* < 0.0001; JF no deprivation vs. JF dep, *p *= 0.02; chow vs. JF dep, *p *= 0.8). Taken with data above, results show that JF increases the number of silent synapses and suggest that the insertion of CP-AMPARs following JF deprivation may then lead to a reduction in silent synapses.

**Fig. 4:**
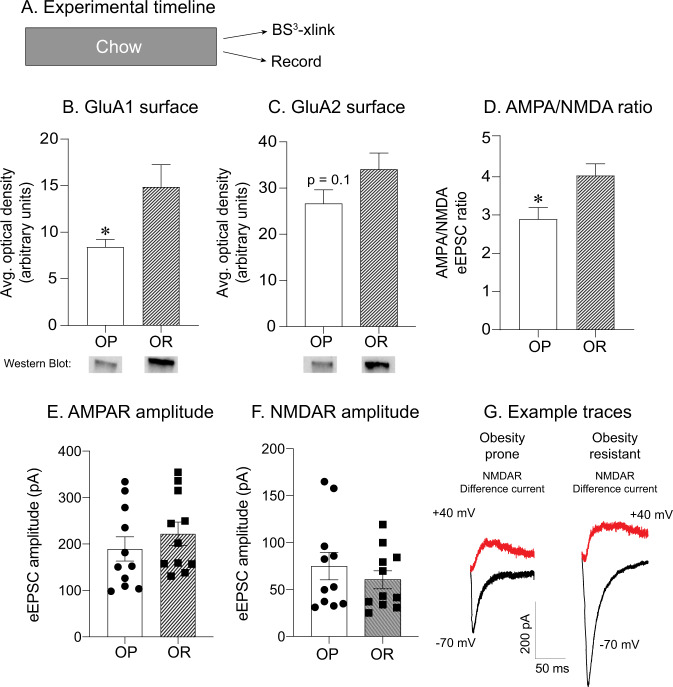
Basal differences in GluA1 surface expression and AMPA/NMDA ratio in obesity-prone vs. obesity-resistant male rats. **a** Experimental timeline. **b** Average GluA1 surface expression in chow fed obesity-prone (OP) and obesity-resistant (OR) male rats. Obesity-prone rats have lower GluA1 surface expression in the NAc compared with obesity-resistant rats. **c** Average GluA2 surface expression in chow fed obesity-prone and obesity-resistant male rats. GluA2 surface expression was similar between obesity-prone and obesity-resistant groups. **d** Average AMPA/NMDA ratio was smaller in obesity-prone vs. obesity-resistant male rats. **e** AMPAR amplitude was similar between obesity-prone and obesity-resistant male rats. **f** NMDAR amplitude was similar between obesity-prone and obesity-resistant male rats. **g** Representative traces of AMPAR-mediated (black) and NMDAR-mediated (red) currents. *p < 0.05 OP vs. OR.

In order to provide clues as to why junk-food produces glutamatergic plasticity in obesity-prone, but not obesity-resistant males, we conducted additional analyses comparing GluA1 and GluA2 expression and excitatory transmission between obesity-prone (*n* = 8) and obesity-resistant (*n* = 9) male controls. Interestingly, GluA1 surface expression was lower in obesity-prone vs. obesity-resistant rats maintained on chow (Fig. 4b: two-tailed unpaired *t*-test, *t*_(15)_ = 2.44, *p* = 0.03), while GluA2 surface expression was similar between these groups (Fig. 4c, *p *= 0.13). This may suggest that basal levels of CP-AMPARs are lower in obesity-prone rats, allowing for greater increases following JF diet.

Furthermore, we also observed a smaller AMPA/NMDA ratio in obesity-prone (*n* = 11 cells, eight rats) vs. obesity-resistant rats (*n* = 11 cells, seven rats; Fig. 4d: two-tailed unpaired *t*-test, *t*_(20)_ = 2.67, *p* = 0.01), indicative of lower levels of excitatory synaptic transmission in obesity-prone groups. Thus, lower basal levels of excitatory transmission in obesity-prone rats may enhance their capacity for subsequent diet-induced plasticity (see “Discussion”). Furthermore, the distribution of raw AMPAR or NMDAR amplitudes was similar across cells from obesity-prone and obesity-resistant rats (Fig. 4e). This suggests that differences in titration of stimulation intensity are not preventing the detection of group differences. Example traces for NMDAR difference current are shown in Fig. 4g.

### Effects of junk-food diet on AMPAR protein expression and synaptic transmission in females

Effects of JF diet on AMPAR subunit expression and excitatory synaptic transmission were also examined in obesity-prone (*n* = 19) and obesity-resistant (*n* = 17) female rats. For these studies we focused on the 2-week deprivation time point because we first wanted to determine if there are persistent effects of JF in females, and because this is when effects had been established in males [39].

**Fig. 5:**
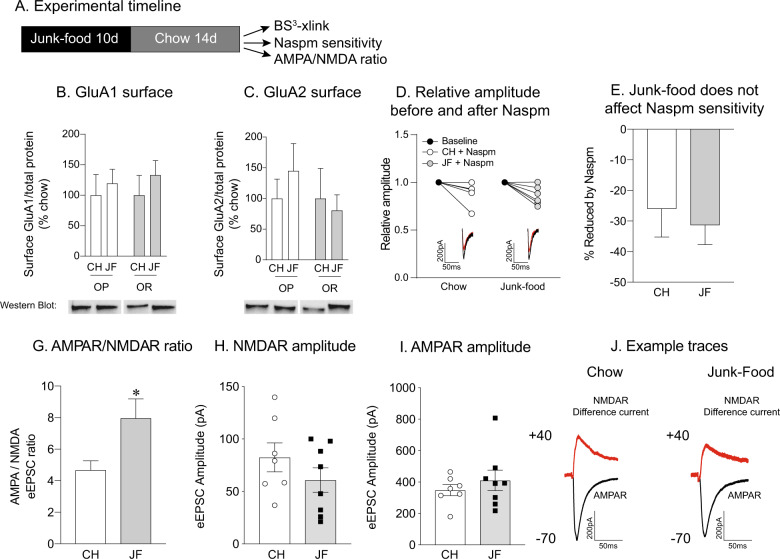
Junk-food does not alter CP-AMPARs expression or function in female rats. **a** Experiment timeline. **b** Average GluA1 surface expression was not affected by junk-food in obesity-prone (OP) or obesity-resistant (OR) female rats. **c** Average GluA2 surface expression was not affected by junk-food in obesity-prone or obesity-resistant female rats. **d** Normalized amplitude before and after Naspm (200 µM): Naspm decreases relative amplitude in both obesity-prone female rats fed a chow and junk-food groups. Inset: example eEPSC from chow and junk-food obesity-prone female groups before (black) and after Naspm (red). **e** No differences in Naspm sensitivity were found between obesity-prone chow vs. junk-food groups. **g** Average AMPA/NMDA ratio was enhanced in obesity-prone females fed a junk-food diet compared with obesity-prone females fed a chow diet. **h** NMDAR amplitude was similar between obesity-prone females fed a chow diet and a junk-food diet. **i** AMPAR amplitude was similar between obesity-prone females fed a chow diet and a junk-food diet. **j** Representative traces of AMPAR-mediated (black) and NMDAR-mediated (red) currents.

Surprisingly, there was no effect of JF on GluA1 (Fig. 5b) or GluA2 (Fig. 5c) surface expression in females following 10 days of JF exposure and 2 weeks of deprivation. It is possible that 10 days of JF exposure was insufficient to trigger plasticity in females. Therefore, we also measured GluA1 and GluA2 surface expression following 30 days of JF diet exposure and 2 weeks of deprivation in obesity-prone females. However, extending the duration of JF exposure to 30 days did not affect GluA1 or GluA2 surface expression in obesity-prone or obesity-resistant female rats (data not shown; GluA1 Surface: two-way ANOVA no diet × group interaction: *F*_(1,24)_ = 1.4, *p *= 0.2; CH vs. JF: *F*_(1,24)_ = 0.4, *p *= 0.5; OP vs. OR: *F*_(1,24)_ = 2.2, *p *= 0.2; GluA2 Surface: two-way ANOVA no diet × group interaction: *F*_(1,27)_ = 1.3, *p *= 0.3; CH vs. JF: *F*_(1,27)_ = 0.5, *p *= 0.5; OP vs. OR: *F*_(1,27)_ = 0.1, *p *= 0.8). Given that effects on surface protein expression and synaptic transmission are not always parallel (e.g., results in males above), we also determined the contribution of CP-AMPARs to NAc core synaptic transmission following 2 weeks of JF deprivation in obesity-prone females (chow: six cells, five rats; JF: six cells, five rats). Consistent with biochemical data, JF did not alter Naspm sensitivity in obesity-prone females (Fig. 5e: two-tailed unpaired *t*-test, *t*_(10)_ = 0.5, *p* = 0.6). To determine effects on excitatory transmission more generally, we also measured the AMPA/NMDA ratio in MSNs from obesity-prone females (chow: *n* = seven cells, five rats; JF: *n* = eight cells, five rats). We found that an increase in the AMPA/NMDA ratio in JF fed vs. chow fed controls (Fig. 5g: two-tailed unpaired *t*-test, *t*_(13)_ = 2.32, *p* = 0.04). However, this effect may be driven by decreases in NMDAR-mediated eEPSC amplitude (Fig. 5h) following JF deprivation, and not overt alterations in AMPAR-mediated transmission were apparent (Fig. 5i). A representative trace of the NMDA difference curve is depicted in Fig. 5j.

In addition, there were no differences in surface or intracellular GluA1 or GluA2 protein expression between obesity-prone and obesity-resistant females fed a chow diet (data not shown; GluA1: surface *p *= 0.9; intracellular *p *= 0.45: GluA2: surface *p *= 0.9; intracellular *p *= 0.11). Thus, results in females differed dramatically from those in males, with no evidence of JF altering AMPAR-mediated transmission (CP-AMPARs or non-CP-AMPARs) or AMPAR subunit protein expression.

## Discussion

There are established roles for NAc core CP-AMPARs in cue-triggered motivation for both food and drug [11, 12, 25]. In addition, increases in NAc CP-AMPAR expression (mixed NAc core and shell) and function (core) can be induced by both cocaine and JF [11, 13, 40]; see also “Introduction”). However, the precise nature of these alterations and the degree to which they may differ between cocaine and JF is not well understood. This has important implications for understanding adaptive vs. maladaptive plasticity that drives food- and drug-seeking behaviors. Furthermore, studies to date have been conducted in males, leaving a large gap in our understanding of potential effects in females. Therefore, here we determined the time course of CP-AMPAR upregulation following JF consumption (with and without a period of JF deprivation) by measuring surface expression of AMPAR subunits and CP-AMPAR-mediated synaptic transmission within the NAc of males and females. In addition, we asked whether CP-AMPAR upregulation in males is accompanied by plasticity of NAc silent synapses. These results are discussed in light of the established time course of NAc CP-AMPAR and silent synapse plasticity produced by cocaine.

### Effects of JF in males

We previously established that eating JF enhances NAc GluA1 but not GluA2 surface expression following 14 or 28 days of JF deprivation, and that CP-AMPAR synaptic transmission is enhanced in the NAc core after 1, 14, or 21 days of JF deprivation in males. However, potential effects on receptor expression at earlier time points and the necessity of a deprivation period for increases in GluA1 protein expression and/or CP-AMPAR synaptic incorporation had not been determined. Here, we found that 10 days of JF consumption produced similar increases in GluA1, but not GluA2, NAc surface expression in obesity-prone males with and without JF deprivation. This pattern is consistent with an increase in the surface expression of CP-AMPARs (either GluA1/GluA1 or GluA1/GluA3 containing). Specifically, the magnitude of increased GluA1 surface expression was similar when tissue was collected after 1 day of JF deprivation or no deprivation period (Fig. 2). Thus, it appears that increases in NAc CP-AMPAR surface protein expression occur during JF exposure (at least by day 10). Furthermore, when tissue was collected following 14 days of JF deprivation, we again found increases in GluA1 but not GluA2, consistent with CP-AMPAR upregulation and previous results [13]. However, the magnitude of this increase was larger than that seen at deprivation day 1. This could suggest that there is a further surface accumulation of CP-AMPARs. Thus, biochemical data indicate that increases in overall surface expression of NAc CP-AMPAR occur during JF exposure (at least by day 10) and persist for relatively long periods after returning to a standard chow diet.

There was variance in weight gain across cohorts used for biochemical and electrophysiological studies (see “Results”). While we cannot rule out potential contributions of weight gain to effects observed here, results to date do not support strong relationships between weight gain and CP-AMPAR increases. Specifically, we find comparable CP-AMPAR increases (both expression and function) whether obesity-prone males are maintained on JF for 10 days or 30 days, and have found that even when food intake and weight gain are the same in obesity-prone vs. obesity-resistant rats CP-AMPAR transmission is increased in obesity-prone but not obesity-resistant males [13]. Furthermore, we have not found any evidence for correlations between weight gain and CP-AMPAR expression of function (unpublished observations based on aggregate data across studies using the same conditions). Thus, we are reasonably confident that differences in weight gain across experiments within the current study are not confounding our results. In addition, Naspm sensitivity in chow fed obesity-prone rats is larger than we have previously reported. A number of factors including titration of stimulation intensity, density of synaptic inputs near recording site, or interexperimenter variance could account for this. However, this does not impact the overall interpretation of our findings as the variance in baseline amplitudes did not differ between experimental groups.

When the effects of JF on NAc core synaptic transmission were examined in males, we found an increase in the contribution of CP-AMPARs after 1 day of JF deprivation compared with chow fed controls (Fig. 3), replicating previous results using this regimen [13]. However, CP-AMPAR-mediated synaptic transmission was similar between chow fed controls and the JF no deprivation group. Thus, although CP-AMPAR surface expression was enhanced with and without JF deprivation (above), a period of JF deprivation was required for the synaptic incorporation of CP-AMPAR in the NAc core. Taken together, data suggest that CP-AMPARs may accumulate extrasynaptically during JF exposure and that JF deprivation is needed to then recruit them to the synapse. There is precedence for the extrasynaptic accumulation of CP-AMPARs following long-access cocaine self-administration [16]; however, whether this is required for increases in synaptic CP-AMPAR transmission is not known.

One notable difference between food vs. cocaine effects is that increases in NAc CP-AMPAR transmission (core) following prolonged cocaine self-administration occur gradually during withdrawal; they are absent 1 day after the last cocaine self-administration session, begin to appear ~14 days later and are markedly increased by ~25 days (see [11] for review). A similar pattern is found for AMPAR subunit surface expression (mixed core and shell, e.g., [15]. Thus, JF consumption has a more rapid effect on NAc CP-AMPAR transmission and expression than prolonged cocaine self-administration. This may not be entirely surprising, given that brain reward circuits evolved in part to be responsive to food and to direct behavior toward needs that are essential for survival. However, it is worthwhile to note that withdrawal from experimenter administered cocaine or short access cocaine self-administration do not produce NAc core CP-AMPAR increases [37, 41, 42], although increases have been found in the shell [43], and a single injection of cocaine is sufficient to increase CP-AMPAR-mediated transmission in the VTA 24 h later [29, 44]. Thus, effects of JF on CP-AMPARs likely vary across regions and type of access.

In sum, CP-AMPAR increases in the NAc core found following JF are more similar to those found following extended access to cocaine self-administration than NAc glutamatergic plasticity following experimenter administered cocaine or short access self-administration. In addition, this upregulation is relatively rapid, and requires a brief period of JF deprivation for CP-AMPAR synaptic incorporation but not increased surface protein expression. One additional consideration for biochemical and electrophysiological studies is that biochemical measures are made from homogenized tissue in samples that contain the NAc core and shell, whereas recordings are made specifically from MSNs in the NAc core. Thus, it is possible that JF produces alterations in protein expression of other types of cells within the NAc, and/or in the NAc shell region.

### Basal differences between obesity-prone and obesity-resistant male rats

It is unclear what triggers and maintains CP-AMPAR synaptic incorporation from a mechanistic standpoint; this is an important and outstanding question in the field [11, 18, 45]. However, basal differences between obesity-prone vs. obesity-resistant males may provide some clues. Specifically, in chow fed controls we found significantly lower basal GluA1 surface expression in obesity-prone vs. obesity-resistant rats (Fig. 4b), without pronounced differences in surface GluA2. This replicates previously established differences in AMPAR subunit surface expression between these strains (see Supplemental Fig. 1 from [3]), and suggests that CP-AMPAR surface expression may be lower in obesity-prone vs. obesity-resistant males. In addition, basal glutamatergic synaptic transmission as measured by the AMPA/NMDA ratio was also lower in obesity-prone vs. obesity-resistant males (Fig. 4d). This reduction appears to be due to a combination of decreased AMPAR transmission and increased NMDAR transmission in the obesity-prone males. Thus, relatively low basal CP-AMPAR surface expression in combination with low basal AMPAR transmission may leave more “room” for the extrasynaptic insertion of CP-AMPARs following JF diet in obesity-prone compared with obesity-resistant males. In addition, the firing threshold of NAc MSNs is lower in obesity-prone vs. obesity-resistant rats [39, 46]. This, in combination with slight elevations in basal NMDAR transmission may facilitate the recruitment of CP-AMPARs to the synapse following JF deprivation.

While basal differences in glutamatergic transmission may help explain why CP-AMPAR surface expression is enhanced following JF consumption, it does not address why deprivation is needed for CP-AMPAR synaptic insertion. As stated earlier, the literature on striatal CP-AMPAR plasticity has overwhelmingly focused on CP-AMPAR upregulation in the incubation of cocaine craving model, with relatively few studies of essential reinforcers, despite strong evidence for incubation of sucrose and high-fat craving [25, 47, 48]. One possibility as to why deprivation is necessary for CP-AMPAR insertion is that junk-food consumption increases in the number of silent synapses which can then undergo subsequent plasticity induced by junk-food deprivation [18, 43]; see below for additional discussion of silent synapses.

### Effects of JF on silent synapses in males

Given the link between CP-AMPARs and plasticity of silent synapses (see “Introduction”), we also determined the proportion of silent synapses in obesity-prone males following JF consumption. There was a marked increase in the proportion of silent synapses following 10 days of JF consumption compared with chow fed controls (Fig. 3e). Furthermore, 1 day of JF deprivation resulted in a return of silent synapses to levels comparable with chow controls. This pattern of an increase followed by a return to baseline in the proportion of silent synapses is consistent with their maturation [29]. In addition, CP-AMPAR insertion itself is associated with the maturation of silent synapses [49]. Thus, these data in combination with CP-AMPAR measures discussed above suggest that JF consumption enhances the number of immature silent synapses, and that the subsequent synaptic insertion of CP-AMPARs following JF deprivation may lead to their maturation. A similar pattern has been found in the NAc shell following cocaine withdrawal although on longer time scales [18].

Increases in silent synapses can be due to the removal of AMPARs from existing synapses, or to the addition of new synapses lacking AMPARs [29, 37, 50]. Our results suggest that JF generates de novo silent synapses. Specifically, we see an increase in GluA1 surface expression in the NAc both with and without deprivation (see Figs. 1a and 2b) and no changes in GluA2 surface expression. If AMPARs were being removed to generate silent synapses from existing synapses, then we would expect to find reductions in GluA1 and/or GluA2 surface protein expression following JF consumption without deprivation. However, this is not the case. In addition, we also saw no evidence for increases in intracellular protein expression, which would be expected to result from AMPAR internalization. Furthermore, high-fat diet consumption increases the density of mature mushroom shaped dendritic spines within the NAc, an indirect indicator of increases in synaptic contacts. Although these spine measures were made after a period of high-fat diet deprivation, they are nonetheless consistent with the idea that consumption of calorie dense foods enhances number of synapses in the NAc.

Although we favor the interpretation that the reduction in silent synapses following JF deprivation is due to maturation of silent synapses because of CP-AMPAR insertion, similarly to cocaine withdrawal (above), there are caveats to the interpretation. First, it is not possible to determine which synapses CP-AMPARs are added to (this is the case for all studies of silent synapses, including past work by TEB, [29, 30]). Second, the inclusion of spermine in our recording pipette could theoretically result in an underestimate of the percentage of silent synapses, particularly when CP-AMPAR-mediated transmission is elevated. Nonetheless, biochemical results in combination with enhanced CP-AMPAR transmission following JF deprivation, an increase in silent synapses following JF consumption alone, and a subsequent reduction in silent synapses after JF deprivation is most parsimonious with a maturation of silent synapses.

### JF does not affect AMPAR expression or function in females

When effects of JF were examined in females, we found no evidence for alterations in AMPAR expression or function. We began with biochemical studies in which we varied the duration of JF exposure, collecting NAc tissue (mixed core and shell as in males) after 10 days or 1 month of JF access and 2 weeks of deprivation. There were no differences in surface or intracellular GluA1 or GluA2 expression in any group. It is possible that JF could have produced transient effects that returned to levels comparable with that of chow controls within the 2-week deprivation period. However, we chose this time point in order to identify effects that persist for reasonably long periods following JF removal, and that correspond to the timing of effects found in males.

Because we did not attempt to control for the cycle in biochemical studies, and ovarian hormones can affect NAc glutamatergic transmission [51], it is possible that variance across the cycle could have masked potentially small effects. Therefore, for electrophysiological studies, recordings were made from slices prepared when females were in metestrus/diestrus. This phase of the cycle was chosen because cue-triggered food seeking and food intake are greatest in this phase [34]. Despite controlling for the cycle, we still found no evidence for JF induced alterations in the CP-AMPAR-mediated transmission (Fig. 5e). Furthermore, although there was an increase in the AMPA/NMDA ratio following 10 days of JF exposure and 2 weeks of deprivation, this may be due to a reduction in the NMDAR mediated component (Fig. 5g–j). Thus, JF induced plasticity differs quite dramatically in males and females and future studies will examine the effect of JF on NMDAR plasticity in females.

To our knowledge this is the first examination of diet-induced NAc glutamatergic plasticity in females, and studies of CP-AMPARs following cocaine exposure have yet to be completed in females. In addition, there is surprisingly little information regarding NAc glutamatergic plasticity in females. However, one recent study found that estradiol applied to slices can alter NAc core mEPSC frequency and amplitude [51], and another showed that estradiol reduces NAc dendritic spine density (an indirect measure of excitatory synapses; [52]). There is however a strong precedence for enhanced sensitivity of females to psychostimulant drugs including cocaine, as well as direct acting dopamine agonists (see [53, 54] for review), and established fluctuations in the preference for food, sex, or drugs across the cycle [34, 54]. Repeated cocaine treatment produces more robust increases in dendritic spine density [55, 56] and mEPSC frequency without concomitant changes in paired pulse facilitation [57] in the NAc of females vs. males. From these studies, Wissman et al. concluded that effects of cocaine on mEPSC frequency in females “reflect differences in excitatory synapse number per neuron rather than presynaptic release probability” [57]. Thus, there is precedence for NAc core glutamatergic plasticity in females following cocaine. However, the absence of effects of sugary, fatty, food here suggest that NAc glutamatergic plasticity differs dramatically between cocaine and essential reinforcers in females. This may not be entirely surprising given the strong influence of ovarian hormones on feeding, metabolism, and energy storage [32, 33] and certainly warrants further investigation.

### Summary and additional considerations

We found that JF consumption increases CP-AMPAR surface expression and generates silent synapses in the NAc of male rats. In addition, a brief period of JF deprivation is needed for the synaptic insertion of CP-AMPARs and the maturation of silent synapses in males. In contrast, JF did not induce AMPAR plasticity in females but may instead alter NMDAR-mediated transmission. Thus, these studies reveal sex differences in the effects of JF on NAc synaptic plasticity. In addition, they provide novel insights into how essential food rewards alter NAc function.

## Funding and disclosure

This work was supported by NIDDK R01DK106188 and NIDDK 1R01DK115526-01 to CRF; TEB was supported by NIDA R01DA040965; YA-C was supported by R01DK106188-02-S1 and 1F99NS108549-01; TLF was supported by NIDA T32DA007268 and R01DK106188; and ETJ was supported by NIDA R01DA040965. The authors declare no competing interests.

## Supplementary information

Supplemental Tables Caption

Supplemental Table 1

Supplemental Table 2
