# Supplementary material for: Sex specific effects of “junk-food” diet on calcium permeable AMPA receptors and silent synapses in the nucleus accumbens core
Source: Neuropsychopharmacology. 2020 Jul 30;46(3):569–78. doi: 10.1038/s41386-020-0781-1 (PMC8027187; doi:10.1038/s41386-020-0781-1)
Supplement: Supplementary file 1 — Supplemental Tables Caption [file 41386_2020_781_MOESM1_ESM.docx]

**Supplemental Table 1. Cumulative weight gained (in grams) across biochemical and electrophysiological experiments. A)** Cumulative weight gained for male and female obesity-prone (OP) and obesity-resistant (OR) rats during the biochemical experiments. **B)** Cumulative weight gained for obesity-prone male and female rats during the electrophysiological experiments.

**Supplemental Table 2. Average food intake (kilo calories [Kcal] consumed) across biochemical and electrophysiological experiments. A)** Average Kcal consumed by male and female obesity-prone (OP) and obesity-resistant (OR) rats during the biochemical experiments. **B)** Average Kcal consumed by obesity-prone male and female rats during the electrophysiological experiments.
