# Supplementary material for: Sex specific effects of “junk-food” diet on calcium permeable AMPA receptors and silent synapses in the nucleus accumbens core
Source: Neuropsychopharmacology. 2020 Jul 30;46(3):569–78. doi: 10.1038/s41386-020-0781-1 (PMC8027187; doi:10.1038/s41386-020-0781-1)
Supplement: Supplementary file 3 — Supplemental Table 2 [file 41386_2020_781_MOESM3_ESM.docx]

^[[1]](#footnote-1)^

1. Missing data for one rat per group [↑](#footnote-ref-1)
