# Supplementary material for: Sex specific effects of “junk-food” diet on calcium permeable AMPA receptors and silent synapses in the nucleus accumbens core
Source: Neuropsychopharmacology. 2020 Jul 30;46(3):569–78. doi: 10.1038/s41386-020-0781-1 (PMC8027187; doi:10.1038/s41386-020-0781-1)
Supplement: Supplementary file 3 [file 41386_2020_781_MOESM2_ESM.docx]

A. Biochemistry: Effects of junk-food on GluA1/GluA2 surface expression in OP and OR rats.					Cumulative weight gained (grams)	Two-way ANOVA			
Experiment	*Sex*	*Strain*	*Diet*	*N*	*Mean ± SE*	*Interaction*	*OP vs. OR*	*CH vs. JF*	*post-test*
"Junk-food" increases NAc GluA1 surface expression only in obesity-prone male rats.	M	OP	CH	10	102 ± 7.3	p = 0.3	p = 0.1	p = 0.4	no group x diet interaction
			JF	10	101 ± 7				
		OR	CH	8	79 ± 1.6				
			JF	12	96 ± 11				
"Junk-food" deprivation is not necessary for increased GluA1 surface expression in obesity-prone male rats.	M	OP	CH	10	91 ± 4	*p = 0.02	*p < 0.0001	p = 0.7	OP CH vs. OP JF: $t_{(56)} = 1.4$, p = 0.3
			JF	20	98 ± 3.5				
		OR	CH	10	77 ± 5.2				
			JF	20	67 ± 1.6				
Effects of "junk-food" diet on AMPAR protein expression in female rats.	F	OP	CH	10	41 ± 7.3	p = 0.3	*p = 0.005	p = 0.5	no group x diet interaction
			JF	10	51 ± 7.2				
		OR	CH	8	30 ± 2.4				
			JF	12	27 ± 3.5				

B. Electrophysiology: Effects of junk-food on CP-AMPAR function and silent synapses in OP rats.					Unpaired t-test			
					Mean ± SE	*t*	*df*	*p-value*
"Junk-food" deprivation is required for increased synaptic CP-AMPAR mediated transmission in obesity-prone male rats.	M	OP	CH	6	38 ± 12	4.7	17	*p = 0.0002
			JF	13	89 ± 4.9			
Generation of silent synapses after "junk-food" exposure	M	OP	CH	6	37 ± 6.4	2.8	13	*p = 0.02
			JF	9	60.9 ± 5.5			
Effects of "junk-food" diet on AMPAR synaptic transmission in females.	F	OP	CH	5	93 ± 15	0.2	8	p = 0.83
			JF	5	89 ± 9			
